# Assessing sleep-related breathing disorders among newly diagnosed rheumatoid and psoriatic arthritis patients: a cross-sectional study

**DOI:** 10.1007/s00296-024-05610-8

**Published:** 2024-05-07

**Authors:** Simon M. Petzinna, Lone Winter, Dirk Skowasch, Carmen Pizarro, Marcel Weber, Daniel Kütting, Charlotte Behning, Claus-Jürgen Bauer, Valentin S. Schäfer

**Affiliations:** 1 Department of Rheumatology and Clinical Immunology, Clinic of Internal Medicine III, University Hospital Bonn, Bonn, Germany; 2 Clinic of Internal Medicine II, University Hospital Bonn, Bonn, Germany; 3 Department of Radiology, University Hospital Bonn, Bonn, Germany; 4 Institute of Medical Biometry, Informatics and Epidemiology, University Hospital Bonn, Bonn, Germany; 5 Department of Rheumatology and Clinical Immunology, Clinic of Internal Medicine III, University Hospital of Bonn, Venusberg-Campus 1, 53127 Bonn, Germany

**Keywords:** Rheumatoid arthritis, Psoriatic arthritis, Sleep apnea syndromes, Sleep-disordered breathing, Sleep Hypopnea, Obstructive sleep apnea, Sleep monitoring

## Abstract

**Objectives:**

This cross-sectional study aimed to determine the prevalence and risk factors for sleep-related breathing disorders (SRBD) in newly diagnosed, untreated rheumatoid arthritis (RA) and psoriatic arthritis (PsA) patients, and to develop a screening algorithm for early detection.

**Methods:**

We evaluated newly diagnosed RA or PsA patients using the Epworth Sleepiness Scale (ESS) questionnaire, cardiorespiratory polygraphy (RPG), and clinical and laboratory assessments. Sleep apnea syndrome (SAS) was diagnosed based on pathological RPG findings excessive daytime sleepiness, defined as ESS score above 10.

**Results:**

The study included 39 patients (22 RA, 17 PsA) and 23 controls. In RPG, SRBD was identified in 38.5% of arthritis patients compared to 39.1% of controls (*p* = 1.00), with male gender (*p* = .004) and age (*p* < .001) identified as risk factors. Excessive daytime sleepiness was noted in 36.4% of RA patients, 17.6% of PsA patients, and 21.7% of controls. Of the 24 patients diagnosed with SRBD, 41.6% met the criteria for SAS. SAS prevalence was 31.8% among RA patients, 0% in PsA patients, and 13% in controls. A significant association was observed between excessive daytime sleepiness and SRBD (*p* = .036).

**Conclusion:**

Our findings reveal a high prevalence of SRBD in newly diagnosed, untreated RA and PsA patients in ESS and RPG, with excessive daytime sleepiness being a reliable predictor of SRBD. Patients with RA exhibited a higher predisposition to SAS. We therefore suggest incorporating ESS and RPG as screening tools in RA or PsA for early detection and management of SRBD.

**Supplementary Information:**

The online version contains supplementary material available at 10.1007/s00296-024-05610-8.

## Introduction

Sleep disorders, which include conditions like sleep-related breathing disorders (SRBD), insomnia, circadian rhythm disturbances, hypersomnia/narcolepsy, parasomnias, and restless legs syndrome disrupt normal sleep patterns, impairing quality of life and emotional health. This can negatively affect physical, mental, and emotional well-being, thereby compromising overall health and safety [1–3].

In the context of arthritis diseases such as rheumatoid arthritis (RA) and psoriatic arthritis (PsA), there is a well described association between sleep disorders and the overall well-being of patients [3–5]. The presence of one can amplify the symptoms of the other. For instance, inadequate disease management leading to joint pain can negatively affect sleep. Conversely, sleep disorders can reduce pain tolerance, amplifying arthritis-associated manifestations [6–9].

Previous studies have shown that a substantial fraction of RA patients, estimated between 40 and 70%, report sleep disturbances, with a higher incidence among men and the elderly [9–11]. Main contributors to these sleep disturbances include pain and functional disability [12, 13]. Similarly, PsA patients often face sleep disturbances, which subsequently contribute to fatigue, anxiety, and depression, negatively affecting their quality of life [4, 14–16]. It’s emphasized that in the case of PsA, sleep disturbances are more often linked to musculoskeletal inflammation than dermatological symptoms [7, 17, 18]. Previous research found that while 16% of individuals with psoriasis experience seep disturbances, this number increased to up to 45% for PsA patients [19, 20]. Across various studies, t’s widely agreed that pain, fatigue, and physical impairments are the major contributors to sleep disturbances in PsA patients, with pain being the predominant factor [4, 7, 20]. However, it’s important to mention that the effects of sleep disturbances on quality of life in RA or PsA patients are often underestimated by healthcare professionals [11, 15].

While numerous studies have explored sleep quality and disturbances in RA or PsA patients with subjective self-report studies, data on SRBD remains sparse. Sleep apnea, characterized by intermittent breathing interruptions during sleep, can stem from upper airway obstruction or insufficient central respiratory center stimulation [21, 22]. This can result in lowered oxygen saturation, frequent arousal, and the subsequent release of stress hormones, thereby leading to nocturnal and daytime symptoms [21, 22]. Such disruptions could significantly impair health, exacerbating daytime sleepiness and reducing physical and mental performance, while elevating cardiovascular morbidity and mortality risks [23, 24].

In light of evidence suggesting that patients with arthritis, particularly those diagnosed with RA and PsA, are at a heightened risk for sleep apnea [25], our study sought to delve deeper into this connection. Previous research has shown varying prevalence rates of sleep apnea within the RA patient population, ranging from 10% to 50% [26–28, 10, 9], with a meta-analysis revealing a prevalence rate of 29.8% among RA patiens [29]. Conversely, data on PsA patients remain scarce, though some studies have pointed to a potential link between sleep apnea and an increased risk of developing PsA or psoriasis [30, 31], while others have found no significant association between PsA and an elevated risk of sleep apnea [32]. By focusing on RA and PsA, our research aims to fill a critical knowledge gap within the field of rheumatology and sleep medicine, while also acknowledging the broader spectrum of spondyloarthritis as an area for future exploration.

This study investigates the prevalence of SRBD in newly diagnosed and untreated RA or PsA patients. Utilizing a comprehensive approach, we incorporated Epworth Sleepiness Scale (ESS) questionnaire to evaluate daytime sleepiness alongside cardiorespiratory polygraphy (RPG) for empirical sleep assessment. Our aim was to establish SRBD screening protocols, enabling physicians to recognize early indicators, enhance patient outcomes, and bridge the existing knowledge gap surrounding SRBD in RA and PsA.

## Materials and methods

### Patient characteristics

Newly diagnosed and untreated RA or PsA patients from the the outpatient clinic of the Department of Rheumatology at the University Hospital Bonn, Germany, were enrolled between August 1, 2018, and August 31, 2022. Diagnosis was made by a board-certified rheumatologist. Inclusion criteria were: American College of Rheumatology (ACR)/ European League Against Rheumatism (EULAR) criteria of 2010 for diagnosis of RA [33], and GRAPPA Recommendations of 2016 for PsA [34]. Eligible patients were also required to have the necessary physical and mental capacity for study participation, be aged over 18, and provide consent for participation. Exclusion criteria included the use of any rheumatological or immunosuppressive medications prior to enrollment. Control participants were recruited from the same department, with a requirement of having no history of rheumatological conditions. Furthermore, each control was thoroughly assessed to confirm the absence of any rheumatological disease at the time of their inclusion in the study. To ensure comparability and reduce potential confounding factors, we performed group matching of these control individuals with our patient cohort based on age and gender. For each patient, demographic data and disease history were documented. The duration of arthritic symptoms and the Disease Activity Score in 28 joints using CRP (DAS28CRP) were recorded by the treating rheumatologist. Smoking history, pre-existing pulmonary conditions, medications, and current symptoms were assessed.

### Cardiorespiratory polygraphy

All patients were tested for sleep apnea using RPG. Board-certified pulmonologists from the University Hospital Bonn interpreted the RPG results, applying criteria adapted from the 2012 American Academy of Sleep Medicine (AASM) guidelines for sleep and associated event assessments. The Apnea-Hypopnea Index (AHI), displaying the number of apneas and hypopneas per hour of total sleep time, was used [35]. An AHI exceeding 5 events/h was considered pathological and indicative of SRBD. SRBD severity levels were assessed as follows: AHI 0–5 events/h: non-pathological; AHI: 5–15 events/h: mild SRBD; AHI > 15–30 events/h: moderate SRBD; AHI > 30 events/h: severe SRBD. When SRBD was diagnosed, further differentiation into obstructive or central sleep apnea was performed. If RPG findings did not allow a clear distinction, the condition was termed nondifferentiable sleep apnea. The diagnosis of sleep apnea syndrome (SAS) required an AHI > 5 events/h and an ESS score exceeding 10. To rule out a cardiological cause for central sleep apnea, transthoracic echocardiography was conducted by a board-certified cardiologist from the University Hospital Bonn.

### Sleep questionnaire

Each patient underwent an assessment using the ESS questionnaire [36]. This scale comprises eight questions with the aim to evaluate the likelihood of an individual dozing off or falling asleep during diverse daily activities. Scores on the ESS can range from 0 to 24. Scores of 10 or below were categorized as normal daytime sleepiness, scores above 10 were considered indicative of excessive, pathological daytime sleepiness.

### Statistical analysis

Exploratory statistical calculations were performed using IBM SPSS 29.0 for Windows. Metric data were presented as mean (± standard deviation (SD)) if normality was assumed and as median (range) for skewed data. In contrast, categorical data were reported as frequencies and percentages. To analyze differences between several groups in terms of metric data, the independent samples t-test or Mann-Whitney U test was used, considering assumptions of normality and homogeneity of variances. To examine differences between binary groups when categorical data were available, the Chi-square test or in the case of small numbers Fisher’s exact test was applied. *P* < .05 was considered statistically significant. Kolmogorov-Smirnov test in conjunction with visual inspection of Q-Q diagrams and histograms were used to evaluate the normality of data distribution. The Levene test of equality of variances was used. Potential confounding factors were controlled by adjusting the inclusion criteria of the control patients according to age and gender. Missing data were handled using a complete case analysis, minimizing the impact of missing data on the results.

### Ethical approval

The study was conducted in accordance with the Declaration of Helsinki and has been reviewed and approved by the ethics committee of the University Hospital Bonn, Germany (reference number: 209/18). Written informed consent was obtained from every patient prior to inclusion in the study. Additionally, ethical considerations regarding the diagnosis and management of sleep-related breathing disorders in arthritis patients were carefully acknowledged and addressed.

## Results

### Patient characteristics

The study prospectively enrolled 39 arthritis patients: 22 with RA and 17 with PsA. Of these, 59.0% (23 patients) were male and 41.0% (16 patients) were female. The control group comprised 23 patients: 60.9% (14 patients) male and 39.1% (9 patients) female. Table 1 presents detailed demographics and characteristics.

### Cardiorespiratory polygraphy

**Fig. 1:**
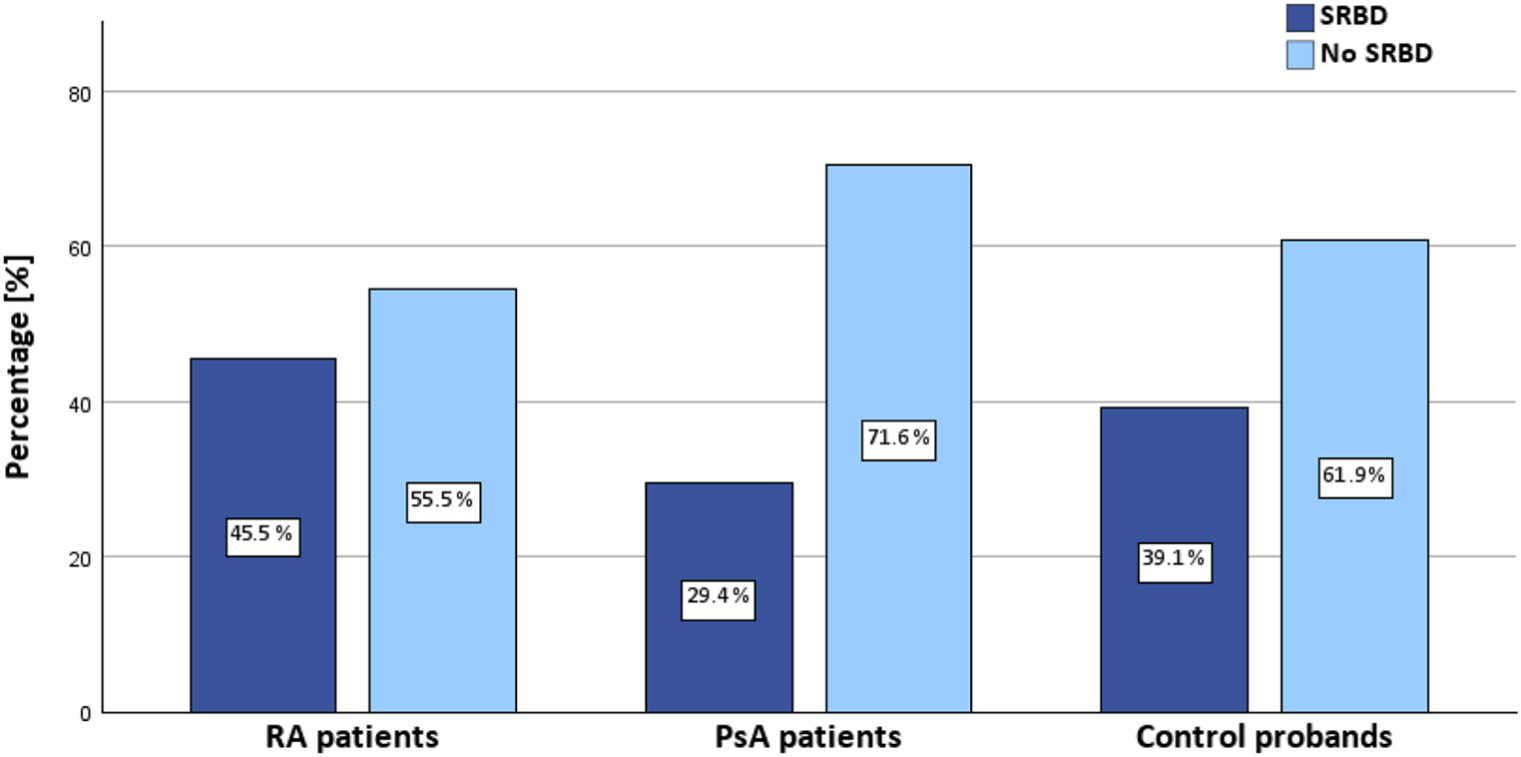
Presence of sleep-related breathing disorders in rheumatoid arthritis patients, psoriatic arthritis patients and control patients: Distribution of sleep-related breathing disorders among patients with rheumatoid arthritis or psoriatic arthritis, and control patients. Within the arthritis cohort, 45.5% of rheumatoid arthritis and 29.4% of psoriatic arthritis patients were diagnosed with sleep-related breathing disorders, compared to 39.1% in the control group; abbrv.: RA: rheumatoid arthritis, PsA: psoriatic arthritis, SRBD: sleep-related breathing disorder

**Fig. 2:**
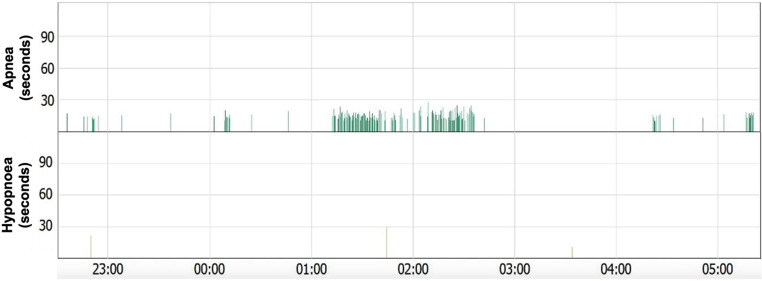
Home sleep apnea testing with cardiorespiratory polygraphy: Exemplary results from cardiorespiratory polygraphy testing

In the cohort of arthritis patients, 15 patients (38.5%) were diagnosed with sleep apnea with an AHI > 5 events/h. This included 45.5% of RA patients (10/22) and 29.4% of PsA patients (5/17). In the control group, 9 individuals (39.1%) exhibited sleep apnea (Fig. 1). Exemplary results from cardiorespiratory polygraphy testing are shown in Fig. 2. Detected risk factors for SRBD include male gender and higher age (Table 1). Comprehensive RPG results are depicted in Table 2. No significant differences were observed between the arthritis and control groups in these findings (*p* > .05). Results from transthoracic echocardiography did not reveal any clinically significant findings or differences related to the presence or absence of SRBD (data not shown).

### Sleep questionnaire

In the cohort of all 62 patients, the median ESS score was 7.5 (range: 0–18). RA patients exhibited a median ESS of 8.0 (range: 0–17), while the median score for PsA patients was 7.0 (range: 4–15). The control group reported a median ESS of 8.0 (range: 0–18). There was no significant association between the presence (RA/PsA patients) or absence (control patients) of rheumatic disease and ESS scores (*p* = .765).

**Fig. 3:**
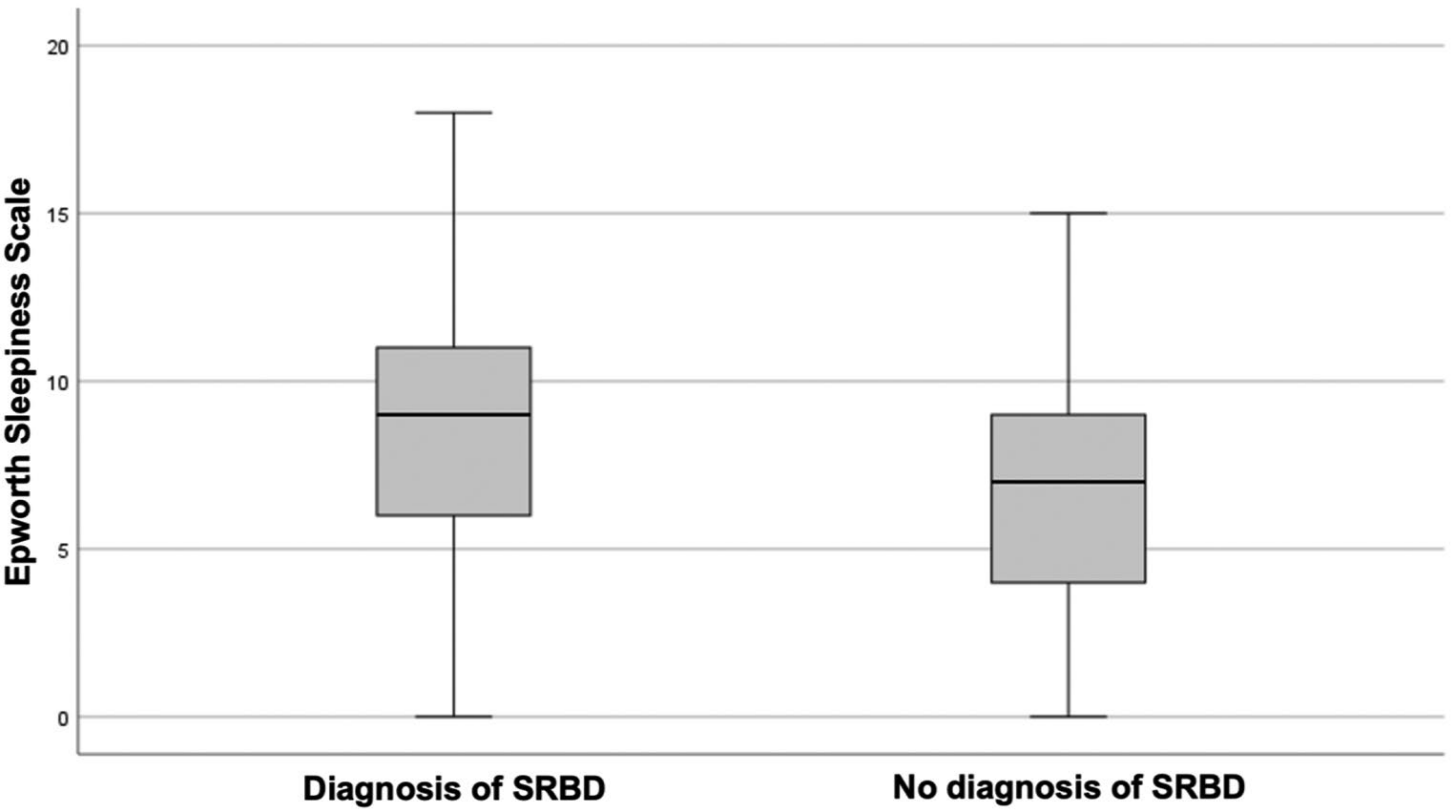
Epworth Sleepiness Scale scores in patients with sleep-related breathing disorders: In patients diagnosed with sleep-related breathing disorder, the median Epworth Sleepiness Scale score was 9.0 (range: 0–18). Patients with non-pathological cardiorespiratory polygraphy exhibited a median Epworth Sleepiness Scale score of 7.0 (range 0–15) (*p* = .132); abbrv.: SRBD: sleep-related breathing disorder

Among patients with SRBD, the median ESS score was 9.0 (range: 0–18). Conversely, those with non-pathological RPG findings exhibited a median ESS score of 7.0 (range: 0–15) (*p* = .132) (Fig. 3). Excessive daytime sleepiness, defined by an ESS score > 10, was identified in 28.2% (11/39) of arthritis patients, 36.4% (8/22) in RA and 17.6% (3/17) in PsA, and in 21.7% (5/23) of controls. Among the 24 patients diagnosed with sleep apnea, 41.6% (10/24) had an ESS score > 10, with a significant difference observed between the subgroups: Seven RA patients, no PsA patient and three control group patients met the criteria for SAS (*p* = .017). A significant association was observed between excessive daytime sleepiness and the presence of SRBD as determined by RPG (*p* = .036).

## Discussion

This study aimed to assess the prevalence of SRBD and associated risk factors in newly diagnosed, untreated RA or PsA patients. To our knowledge, this is the first analysis in this cohort, employing a multimodal approach with the ESS questionnaire for self-reported daytime sleepiness and the RPG for empirical sleep assessment. Furthermore, our objective was to develop a SRBD screening algorhythm for these two diseases, with the goal to improve early detection by clinicians and thereby enhance patient outcomes.

Previous research, largely cross-sectional studies utilizing self-reported questionnaires, have primarily explored sleep disorders among RA and PsA with chronic conditions experiencing general sleep disturbances [9–11, 4, 14, 15]. Musculoskeletal manifestations and associated pain have been identified as primary contributors to disrupted sleep [12, 13]. In our study, the ESS questionnaire was employed to assess these sleep disturbances [36]. No significant deviation in the ESS score was observed when comparing patients with RA or PsA to controls. Excessive daytime sleepiness was evident in 28.2% of arthritis patients (RA:36.4%; PsA: 17.6%) and 21.7% o controls.

The reduced prevalence of sleep disturbances observed in our study relative to existing research on RA [9–11] and PsA [4, 14–16] patients, and the absence of a statistically significant association between sleep disturbance prevalence and diagnosis as indicated by the ESS, may be attributed to our emphasis on investigating newly diagnosed patients. It’s well-established that sleep disturbances in patients with RA or PsA can origin from acute inflammation leading to pain and articular damage [12, 13]. Furthermore, fragmented sleep can exacerbate pain, engendering a vicious cycle [37]. Thus, while acute inflammation with subsequent pain may remain the primary contributor to reported sleep disturbances in our cohort, our focus on patients with first diagnosis excluded the potential contribution of post arthritis joint damage to sleep disturbances. This reiterates the importance of timely diagnosis and intervention in these diseases. With the advancement in therapeutic strategies, which proficiently reduce inflammation, there’s a likelihood that both sleep disturbances due to acute inflammation as well as sleep disturbances tied to joint damage might diminish over time. This could translate to a reduced prevalence of sleep disturbances. Future research would benefit from a longitudinal assessment of ESS questionnaires to track any emerging trends.

However, given the multifaceted nature of sleep disorders, a singular focus on inflammation-associated symptoms, subsequent pain, and self-reported daytime sleepiness falls short of encompassing the full spectrum of potential sleep disturbances in arthritis diseases. Few studies have investigated the prevalence of SRBD among individuals with RA or PsA before [30, 31, 26–28, 10, 9, 29]. A significant knowledge gap persists due to the absence of prospective, observational studies on the prevalence of SRBD in early-stage and newly diagnosed patients. An easily available and applicable screening instrument for SRBD is urgently needed. While polysomnography remains the gold-standart for diagnosing SRBD due to its sensitivity, its application has limits. It is notably time-intensive and requires considerable resources, including specialized expertise for accurate administration, assessment, and interpretation [10]. In contrast, RPG emerges as a more accessible, cost-effective, and expedient alternative, requiring less specialized knowledge for interpretation. RPG has been validated as an effective tool for screening, efficiently identifying individuals at heightened risk of SRBD, in particular suitable for primary care and outpatient settings [38]. Given these considerations, our study opted for the ESS and RPG, prioritizing ease of access, patient comfort, and adherence, as well as the pragmatic applicability in a clinical setting, providing valuable insights into the prevalence and risk factors of SRBD in newly diagnosed and untreated RA or PsA patients.

Employing RPG, our research revealed a 45.5% prevalence of SRBD in RA patients, which partially exceeds the prevalence reported in previous cross sectional studies. For instance, a meta-analysis conducted by Thakur et al. noted a 29.8% prevalence of SRBD among RA patients [29]. Importantly, there has been just one other prospective, albeit cross-sectional, study that has utilized empirical sleep assessment (polysomnography) to ascertain the prevalence of SRBD in RA, finding sleep apnea in 47.7% o the participants [39]. Literature on SRBD prevalence in PsA remains even more limited, with cross-sectional, self-reported sleep assessments being the primary method for examining the link between SRBD and PsA [30, 31]. Our research assesses SRBD among newly diagnosed and untreated patients with PsA, with our findings indicating a 29.4% prevalence ofSRBD in this cohort, the first report in this patient population.

To identify potential risk factors for SRBD in RA or PsA, we examined the relationship between demographic data and RPG findings. Existing literature has identified several risk factors for SRBD, including male gender, advancing age, obesity, smoking habits, and disease ativity [21, 22]. In alignment with these findings, our study indicates male gender and older age as significant risk indicators for SRBD in patients newly diagnosed with RA or PsA. Notably, our investigation found no substantial link between the duration of RA or PsA symptoms and SRBD occurrence. It’s important to recognize, however, that the relatively short median symptom duration of 6 months, as observed in our study group, might not be representative for disease experiences in regular RA and PsA patient.

In contrast, our study suggests possible links between smoking status, disease activity as reflected by DAS28CRP scores and BMI with the occurrence of SRBD, although these associations did not achieve statistical significance. The potential association between increased BMI and SRBD warrants particular focus, especially in the light of the link that recent research has revealed between rheumatological diseases, metabolic syndrome, and sleep apnea [40]. The presence of metabolic syndrome has been associated with a heightened risk of developing RA and PsA, further amplifying the risk of SRBD [41, 42]. Thus, central obesity, insulin resistance, dyslipidemia, and hypertension has been shown to contribute to upper airway obstruction and impaired respiratory function during sleep [43]. The reciprocal relationships between metabolic syndrome, arthritis, and sleep apnea need to be investigated in more detail to possibly decipher synergistic therapeutic approaches. Future large-scale studies are needed to confirm these risk factors for SRBD in newly diagnosed and untreated RA or PsA patients.

Comparing the ESS scores with RPG results, the ESS score was shown to be a reliable predictor for SRBD, revealing an association with RPG-identified SRBD. Moreover, we found that the occurrence of SAS, defined by an ESS score exceeding 10 combined with pathological RPG outcomes, was significantly more prevalent in RA patients than in PsA patients or the control group. Several hypotheses can be discussed regarding the observed epidemiological link between SAS and RA. Elevated levels of proinflammatory cytokines in patients with SRBD such as TNF-α, IL-6, IL-8, may exacerbate systemic inflammatory responses and, consequently, increase the vulnerability to RA [44–47]. Moreover, shared etiological factors such as genetic predispositon, environmental exposures, prevalent comorbidities, and lifestyle determinants might contribute to the noted association between RA and SRBD [48].

In conclusion, our study evaluates prevalence of SRBD in newly diagnosed, untreated RA or PsA patients in a multimodal approach. The ESS questionnaire and RPG have emerged as viable diagnostic tools, revealing a high prevalence of excessive daytime sleepiness and SRBD in this cohort. Moreover, ESS questionnaire serves as a valuable predictor for SRBD and SAS, which were frequently detected in our arthritis patients, particularly those with RA. Clinicians should be alert to the detected key risk factors for SRBD, especially male sex and older age above 55 years, considering the negative impact of SRBD on daily physical and cognitive functions. We advocate for the incorporation of routine ESS questionnaire and RPG screenings, as easy, cost-efficient diagnostic tools for all newly diagnosed RA or PsA patients. Such an approach ensures the timely identification and treatment of SRBD, significantly improving patient outcome.

While our study sheds light on the link between SRBD and RA or PsA, it also underscores the need for additional research due to several acknowledged limitations. One notable constraint is the potential restricted applicability of our results, stemming from single center recruitment of RA and PsA patients. This selection process might not fully represent the diversity of demographic characteristics and disease severity seen across broader populations, possibly influencing the reported prevalence and risk factors for SRBD and introducing selection bias. Furthermore, our study’s relatively modest sample size could diminish the statistical robustness of our findings, suggesting the necessity for future investigations with larger cohorts to validate our observations. Additionally, our analysis might not have comprehensively accounted for all conceivable confounding variables. Critical factors such as comorbid conditions (e.g., diabetes and hypertension), medication usage, socioeconomic factors, symptom duration, and neck circumference were not exhaustively evaluated and were not part of the exclusion criteria, which could compromise the reliability of our conclusions.

Future research should adopt a longitudinal framework to examine the evolution of SRBD following the initiation of treatment for RA or PsA. This perspective would allow for a detailed examination of the reciprocal influences between SRBD and arthritis progression over time. Furthermore, it remains to be clarified whether the sleep disturbances identified in our study are specific to RA and PsA, or if they manifest a common pattern across various forms of arthritis or autoimmune diseases, including the broader category of spondyloarthritis.

### Related Congress Abstract Publication

Parts of this study were presented at the EULAR 2023 congress and have been published in the EULAR abstract supplement of the Annals of the Rheumatic Diseases [49].

**Table 1:** Patient demographics and associated diseases

		Rheumatic diseases			Sleep-related breathing disorder		
		RA (*n* = 22)	PsA (*n* = 17)	Control patients (*n* = 25)	Present (*n* = 24)	Not present (*n* = 38)	p-value
Sex	Female	6	10	14	6	24	0.004*
		27.3%	58.8%	60.9%	25.0%	63.2%	
	Male	16	7	9	18	14	
		72.7%	41.2%	39.1%	75.0%	36.8%	
Age at time of RPG [years]	Median	56.4	44.9	50.8	57.0	42.5	< 0.001*
	Min, Max	21.7, 81.9	20.2, 67.0	25.3, 67.5	36.2, 81.9	20.3, 76.1	
BMI [kg/m^2^]	Median	24.8	25.8	27.8	27.4	25.8	0.112
	Min, Max	18.4, 38.5	18.9, 36.2	17.6, 35.9	22.3, 38.5	17.6, 36.2	
Smoking	Neversmoker	16	8	18	14	28	0.268
		72.7%	47.1%	78.3%	58.3%	73.7%	
	Eversmoker	6	9	5	10	10	
		27.3%	52.9%	21.7%	41.7%	26.3%	
Respiratory symptomatology	Not present	14	12	20	16	30	0.374
		63.6%	70.6%	87.0%	66.7%	78.9%	
	Present	8	5	3	8	8	
		36.4%	29.4%	13.0%	33.3%	21.1%	
Previous pulmonary disease	None	19	16	20	23	32	0.576
		86.4%	94.1%	87.0%	95.8%	86.5%	
	Bronchial asthma	2	1	1	1	3	
		9.1%	5.9%	4.3%	4.2%	8.1%	
	COPD	0	0	0	0	0	
		0.0%	0.0%	0.0%	0.0%	0.0%	
	Sarcoidosis	0	0	2	0	2	
		0.0%	0.0%	8.7%	0.0%	5.4%	
Arthritis symptom duration [months]	Median	4.0	9.0	N/A	4.0	7.0	1.000
	Min, Max	0.1, 38.0	18.9, 36.2	N/A	0.1, 38.0	2.0, 120.0	
CRP [mg/l]	Median	7.7	3.1	1.7	4.8	2.4	0.392
	Min, Max	0.3, 145.4	0.3, 56.8	0.61, 6.0	0.64, 145.4	0.3, 85.3	
DAS28CRP	Median	3.7	3.2	N/A	3.6	3.2	0.214
	Min, Max	2.1, 7.0	2.1, 5.4	N/A	2.1, 7.0	2.1, 6.7	

Details of demographics for rheumatoid arthritis, psoriatic arthritis, and control group patients and sleep-related breathing disorders. C-reactive protein levels were significantly associated with the presence of rheumatic disease. Advanced age and male gender were significantly associated with the diagnosis of sleep-related breathing disorders; abbrv.: RA: rheumatoid arthritis, PsA: psoriatic arthritis, SD: standard deviation, BMI: body mass index, COPD: chronic obstructive pulmonary disease, CRP: C-reactive protein, DAS28CRP: Disease Activity Score in 28 joints using CRP, N/A: not applicable; *p < .05 was considered significant. Statistical tests only compare rheumatic disease group to controls, but not RA vs. PsA vs. controls.

**Table 2:** Cardiorespiratory polygraphy outcomes

		Rheumatic diseases		Control patients (*n* = 25)	p-value
		RA (*n* = 22)	PsA (*n* = 17)		
Presence of a sleep-related breathing disorder	Present	10 45.5%	5 29.4%	9 39.1%	1.000
	Not present	12 54.5%	12 70.6%	14 60.9%	
AHI [events/h]	Median	4.9	1.7	4.1	0.390
	Min, Max	0.3, 35.3	0.0, 40.2	0.3, 32.5	
Apnea Index [events/h]	Median	0.4	0.4	0.7	0.613
	Min, Max	0.0, 23.2	0.0, 28.7	0.0, 27.9	
Hypopnea Index [events/h]	Median	3.6	0.8	3.0	0.461
	Min, Max	0.0, 26.5	0.0, 11.5	0.0, 18.5	
Total obstructive apneas	Median	0.0	0	1.0	0.445
	Min, Max	0.0, 116.0	0.0, 105.0	0.0, 137.0	
Total central apneas	Median	2.0	2.0	1.0	0.709
	Min, Max	0.0, 45.0	0.0, 155.0	0.0, 70.0	
Total combined obstructive-central apneas	Median	0.0	0.0	0.0	0.089
	Min, Max	0.0, 5.0	0.0, 18.0	0.0, 44.0	
ODI [events/h]	Median	7.1	3.1	4.7	0.587
	Min, Max	0.0, 37.2	0.0, 41.0	0.1, 32.4	
Average saturation [%O_2_]	Median	94.4	95.1	92.7	0.368
	Min, Max	87.1, 97.7	90.2, 96.8	90.4, 97.4	

Detailed results from cardiorespiratory polygraphy among rheumatoid arthritis, psoriatic arthritis, and control group patients; abbrv.: RA: rheumatoid arthritis, PsA: psoriatic arthritis, AHI: Apnea-Hypopnea Index, OHI: Oxygen Desaturation Index. *p < .05 was considered significant. Statistical tests only compare rheumatic disease group to controls, but not RA vs. PsA vs. controls.

### Electronic supplementary material

Below is the link to the electronic supplementary material.

Supplementary Material 1

## Data Availability

Data are available on reasonable request.

## Declarations

All authors take full responsibility for the accuracy and integrity of all aspects of the work. The authors report no disclosures relevant to the manuscript.
